# Clinical and Cost-Effectiveness of Procalcitonin Test for Prodromal Meningococcal Disease–A Meta-Analysis

**DOI:** 10.1371/journal.pone.0128993

**Published:** 2015-06-08

**Authors:** Jennifer M. Bell, Michael D. Shields, Ashley Agus, Kathryn Dunlop, Thomas Bourke, Frank Kee, Fiona Lynn

**Affiliations:** 1 Centre for Infection and Immunity, School of Medicine, Dentistry and Biomedical Sciences Queen’s University Belfast, Belfast, Northern Ireland, United Kingdom; 2 Northern Ireland Clinical Trial Unit, Belfast Health and Social Care Trust, Belfast, Northern Ireland, United Kingdom; 3 Ulster Hospital Dundonald, South Eastern Health and Social Care Trust Belfast, Northern Ireland, United Kingdom; 4 Royal Belfast Hospital for Sick Children, Belfast Health and Social Care Trust, Belfast, Northern Ireland, United Kingdom; 5 UKCRC Centre of Excellence for Public Health (NI), School of Medicine, Dentistry and Biomedical Sciences Queen’s University Belfast, Belfast, Northern Ireland, United Kingdom; 6 School of Nursing and Midwifery, Queen’s University Belfast, Belfast, Northern Ireland, United Kingdom; The University of Tokyo, JAPAN

## Abstract

**Background:**

Despite vaccines and improved medical intensive care, clinicians must continue to be vigilant of possible Meningococcal Disease in children. The objective was to establish if the procalcitonin test was a cost-effective adjunct for prodromal Meningococcal Disease in children presenting at emergency department with fever without source.

**Methods and Findings:**

Data to evaluate procalcitonin, C-reactive protein and white cell count tests as indicators of Meningococcal Disease were collected from six independent studies identified through a systematic literature search, applying PRISMA guidelines. The data included 881 children with fever without source in developed countries.The optimal cut-off value for the procalcitonin, C-reactive protein and white cell count tests, each as an indicator of Meningococcal Disease, was determined. Summary Receiver Operator Curve analysis determined the overall diagnostic performance of each test with 95% confidence intervals. A decision analytic model was designed to reflect realistic clinical pathways for a child presenting with fever without source by comparing two diagnostic strategies: standard testing using combined C-reactive protein and white cell count tests compared to standard testing plus procalcitonin test. The costs of each of the four diagnosis groups (true positive, false negative, true negative and false positive) were assessed from a National Health Service payer perspective. The procalcitonin test was more accurate (sensitivity=0.89, 95%CI=0.76-0.96; specificity=0.74, 95%CI=0.4-0.92) for early Meningococcal Disease compared to standard testing alone (sensitivity=0.47, 95%CI=0.32-0.62; specificity=0.8, 95% CI=0.64-0.9). Decision analytic model outcomes indicated that the incremental cost effectiveness ratio for the base case was £-8,137.25 (US $ -13,371.94) per correctly treated patient.

**Conclusions:**

Procalcitonin plus standard recommended tests, improved the discriminatory ability for fatal Meningococcal Disease and was more cost-effective; it was also a superior biomarker in infants. Further research is recommended for point-of-care procalcitonin testing and Markov modelling to incorporate cost per QALY with a life-time model.

## Introduction

Diagnostic tests rarely give a definitive dichotomous result (a diagnosis of disease or no disease) but more often offer results on a continuous likelihood or probability scale. Thus a test threshold is required to indicate the likelihood of disease. With imperfect tests such thresholds are difficult to determine. Avoiding underdiagnoses and ensuring diagnosis of each life threatening case is more important than the costs and consequences attendant upon a falsely positive diagnosis in a healthy person [1].

Meningococcal Disease (MD) is an example of a potentially fatal illness if diagnosis is missed or delayed. The meningococcus invades the thin membrane covering the brain and spinal cord (meningococcal meningitis) or blood (meningococcal sepsis), and often both [2]. MD mostly affects children less than five and young people of 17 to 19 years of age. Vaccines are available to protect against different strains of the microorganisms. The rates of MD have declined. However, serogroup B meningococcal (MenB) disease is the most common cause of MD in older children, young adults in the United States (US)[3] and, recently, across age groups in the UK accounting for 85–90% of cases [4]. In 2009–10 there were around 1000 laboratory-confirmed MD cases in the UK and Ireland [5]. The perception of prognosis for MD now is that few cases result in death probably because of improved intensive care provision. The perceived lower case fatality may paradoxically delay the uptake of vaccines, which must undergo stringent national regulatory authorisation [6]. The Men B vaccine is not approved in the US but it has been used to help control specific outbreaks in the US. The licensed vaccine is not routinely recommended for use in Europe, Canada, and Australia. In March 2014, The Joint Committee on Vaccinations and Immunisation (JCVI) reviewed their decision of insufficient evidence to support routine MenB vaccination for using Bexsero (Novartis Vaccines and Diagnostics S.r.l.) in the UK to recommend a carefully planned vaccination programme for MenB initially in infants [7]. Meanwhile, clinicians must continue to be vigilant regarding possible MD in children and young people of all ages, as, even with constantly improving paediatric intensive care services, more children will survive invasive MD with disability [8,9].

Most paediatric patients with MD make a full recovery but some are left with critical complications [2, 8–11]. NICE has estimated that of the survivors 3% have amputations, 3% have other orthopaedic complications for example damage to growth plates and 13% have skin complications that require reconstructive surgery [12]. A recent UK case-control study into the outcomes of invasive MD (strain MenB) in survivors reported: around 10% of children suffered major disabling deficits (seizures, hearing loss, amputations, visual loss and loss of speech or ability to understand speech) and more than a third had minor deficits (other physical, cognitive, and psychological abnormalities) [9]. These problems severely affect the quality of life experienced by survivors and their families [2]. Adverse outcomes in each survivor of severe MD are estimated to accumulate life-long costs totalling £1.3 million [13]. Early detection and prompt treatment of early stage MD (prodromal stage) can halt disease progression and reduce the severity of the clinical outcome. Prodromal stage MD is extremely difficult to clinically distinguish from less serious illnesses. If not detected early, some MD cases rapidly escalate to a fulminant illness. Results from traditional laboratory blood tests for MD detection are usually not available early enough to influence treatment. It has been estimated that 50% of children presenting to General Practitioners (GPs) in the UK with prodromal MD are misdiagnosed with other conditions and sent home [14]. Conversely, it is well recognised that misdiagnosis of MD also occurs leading to many children needlessly being admitted to hospital and treated with intravenous antibiotics.

Levels of procalcitonin (PCT) in the blood, a naturally occurring hormone, rise within two hours of the onset of an invasive infection, peak at six hours and remain elevated for a further 24 hours. PCT can be detected rapidly and accurately using a near-patient test (B-R-A-H-M-S PCT, ThermoScientific). PCT is not routinely measured in Emergency Departments (EDs) despite its potential usefulness as an early indicator of infection [15–19]. While never completely ruling out MD when combined with a careful clinical assessment, a PCT test could be useful in the assessment of a child with nonspecific fever.

Existing NICE clinical guidelines (CG102) on the management of bacterial meningitis and meningococcal septicaemia in children and young people in the UK do not recommend the use of serum PCT levels for prediction of disease [12]. C-reactive protein (CRP) and white cell count (WCC) measurement is recommended as a potential indicator of bacterial meningitis in febrile children with a rash although these are non-specific indicators of severe infection. Scottish Intercollegiate Guidelines Network (SIGN) acknowledge that serum PCT levels could help distinguish patients with a fever without source (FWS) who have serious bacterial infection (SBI) from those who do not [20]. High PCT levels at the time of hospital admission in children with MD have been associated with the severity of outcome, sepsis and death [21–23]. Thus, based on current evidence, the SIGN guidelines state that clinicians should be aware that a high PCT level (>150ng/l) is associated with high mortality.

PCT is a good indicator of early SBI [24–28]. In this review we wish to establish if the PCT test’s diagnostic performance is better than that of CRP and WCC for the detection of the prodromal stage MD in children presenting at ED with a FWS. However, test performance alone is of little relevance detached from clinical decision-making. To determine the value of using a PCT test as a prompt indicator of prodromal MD in febrile children, we evaluated the test accuracy and cost-effectiveness in plausible clinical scenarios using data from independent studies carried out in developed countries. In addition, an analysis of unpublished results was included from a previous study carried out in Belfast.

## Methods

### Selection Criteria

1. Children aged 1 month to 16 years (as a subgroup) with suspected MD were eligible for inclusion if: they had FWS, were admitted to the ED or initial admissions unit of a hospital in a middle-high income country, had serum PCT tested within 4 hours of arrival and had no previously known bacterial or viral infection. FWS was defined as a temperature greater than 38 degrees Celsius with no apparent source after clinical history and examination.
2. The PCT test employed was a luminescence immunoassay (Brahms Diagnostica GmbH, D-12099 Berlin, Germany) or very similar technology. Studies may or may not have measured CRP and WCC. It was assumed that CRP and WCC analyses were performed in quality assured hospital laboratories. For comparison reasons the blood samples for each test (PCT, CRP and WCC) had to be taken at the same time during initial clinical presentation.
3. MD caused by *Neisseria meningitidis* was confirmed using one of the following ‘gold standard’ tests: conventional culture or polymerase chain reaction (PCR). The blood or cerebrospinal fluid (CSF) used for these tests was taken at the same time as sampling for PCT testing:

### Data Source

To identify all prospective and retrospective studies and Randomised Controlled Trials (RCTs) a literature search, using search terms relating to children, FWS, Meningococcal, bacterial meningitis and PCT was carried out in August 2011 (Table A in S1 File). Ovid MEDLINE(R) and MEDLINE(R) In-Process & Other Non-Indexed Citations, Embase, HMIC Health Management Information Consortium, Web of Knowledge Web of Science, BIOSIS Citation Index, BIOSIS Previews, EBSCO CINAHL Plus and The Cochrane Library inclusive of Cochrane Controlled Trials Register were searched. Additional searches involved ZETOC (general and conference), Index to Thesis of Great Britain and Ireland, Pro-Quest Education Journals, Turning Research into Practice (TRIP), National Library for Health (NLH), e-Guidelines, NICE guidelines, SIGN guidelines and Clinical Trials.gov. Hand-searching of reference lists in relevant articles was carried out.

Abstracts were reviewed and full-text articles were obtained for those studies that met the eligibility criteria. The correspondence authors of relevant studies were contacted to request raw data for PCT and, if available, CRP and WCC to enable calculation of true positives (TP), false positives (FP), false negatives (FN), and true negatives (TN) with MD confirmation by culture or PCR microbiological testing. Each individual study was assessed for quality by using the revised tool for the Quality Assessment of Diagnostic Accuracy Studies (QUADAS-2) (S1 PRISMA Checklist)

### Statistical analysis

PCT, CRP and WCC levels for cases of microbiologically confirmed MD or not MD seen in ED or on admission were analysed per study and within a combined studies pooled data set. Receiver operator curve (ROC) analysis of pooled study data was used to compare diagnostic performance of each test from the areas under the curve (AUC) with 95% confidence levels (CIs). Optimal diagnostic test cut-off levels were found using the Youden Index. For each test, 2 x 2 tables were constructed using optimal diagnostic test cut-off levels.

Meta-analyses, employing the random-effects and Hierarchical Summary ROC models (HSROC), were carried out to demonstrate relative risk (RR) for the studies using the optimal diagnostic test thresholds for early MD. The summary threshold for each test was identified on the HSROC curve for each test. Subgroup meta-analysis of test performance was undertaken for different age-groups. Statistical analysis was undertaken using Stata/IC software version 11 (StataCorp, College Station, Texas).

#### Assessment of heterogeneity

To quantify the extent of between study variations (heterogeneity), the I^2^ Statistic was calculated prior to meta-analysis. Publication bias was explored using Egger’s test and funnel plots.

### Cost-effectiveness analysis

**Fig 1:**
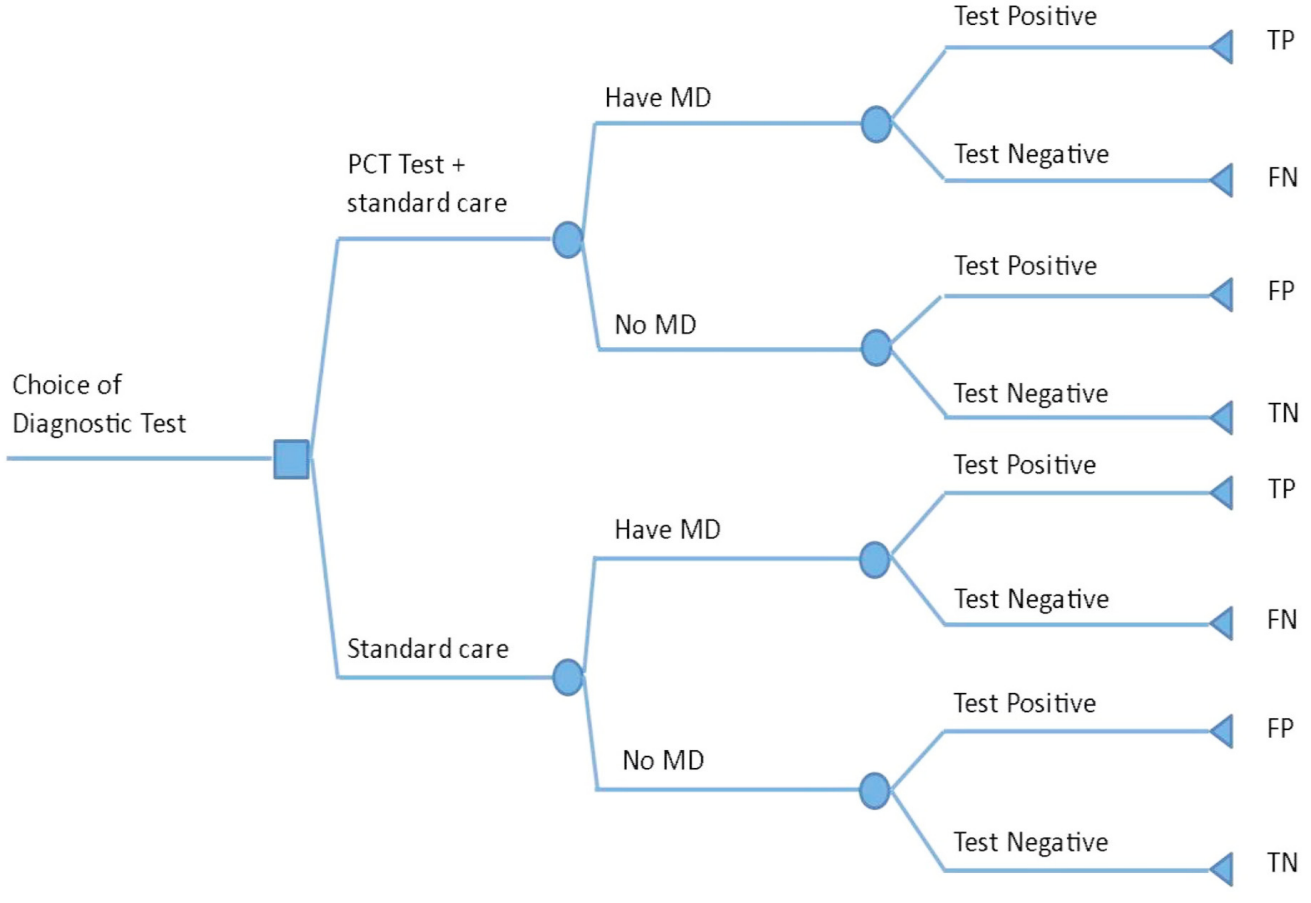
Decision tree for clinical pathway for children with fever without source.

A decision analytic model was constructed to establish the cost-effectiveness of PCT, CRP and WCC tests compared with CRP and WCC tests (standard care) in the diagnosis of MD in children presenting at ED with a FWS. Fig 1 presents a simplified illustration of the decision tree. The model makes a clear link between the diagnostic accuracy of a given test, the impact on treatment decisions and the ultimate effect on correct treatment and costs [29]. The cost-effectiveness analysis was conducted from the perspective of the National Health Service (NHS) and only included hospital costs associated with the diagnosis and follow-up. Clinical pathways were detailed for four diagnostic groups (i.e. those with true positive results, false negative results, true negative results and false positive results) with a further pathway to represent the level of illness (severe, moderate or mild). The probability distributions relating to the four diagnostic groups were derived from the meta-analysis; while the distributions for the severity of illness were based on data from a single source [30]. Costs and outcomes of each of the groups were assessed. Multiple clinical pathways were based on an outcomes study among children with suspected MD assessed at the RBHSC [30] and costed in UK Sterling (£) using unit costs from the National Schedule of Reference Costs 2010–2011 of NHS Trusts and Primary Care Trusts combined [31] (Tables D and E in S1 File). The unit costs for hospital spells do not include costs associated with paediatric critical care (PICU). These costs were therefore added separately. The tests were assumed to be carried out on the same blood sample collected from the ill child on presentation to the ED. No discounting of costs and health outcomes were applied as the time horizon was less than 1 year. The cost-effectiveness was expressed in terms of the incremental cost-effectiveness ratio (cost per correctly treated patient). A correct diagnosis was defined as either a true positive or a true negative result.

#### Sensitivity analysis

One-way sensitivity analysis was used to explore the impact of alternative assumptions and to assess the effect of uncertainty. The analysis was performed by altering the diagnostic thresholds for each test option (derived from the summary HSROC statistics) with the effects of these changes on the incremental cost-effectiveness ratios subsequently derived.

## Results

### Study identification

**Fig 2:**
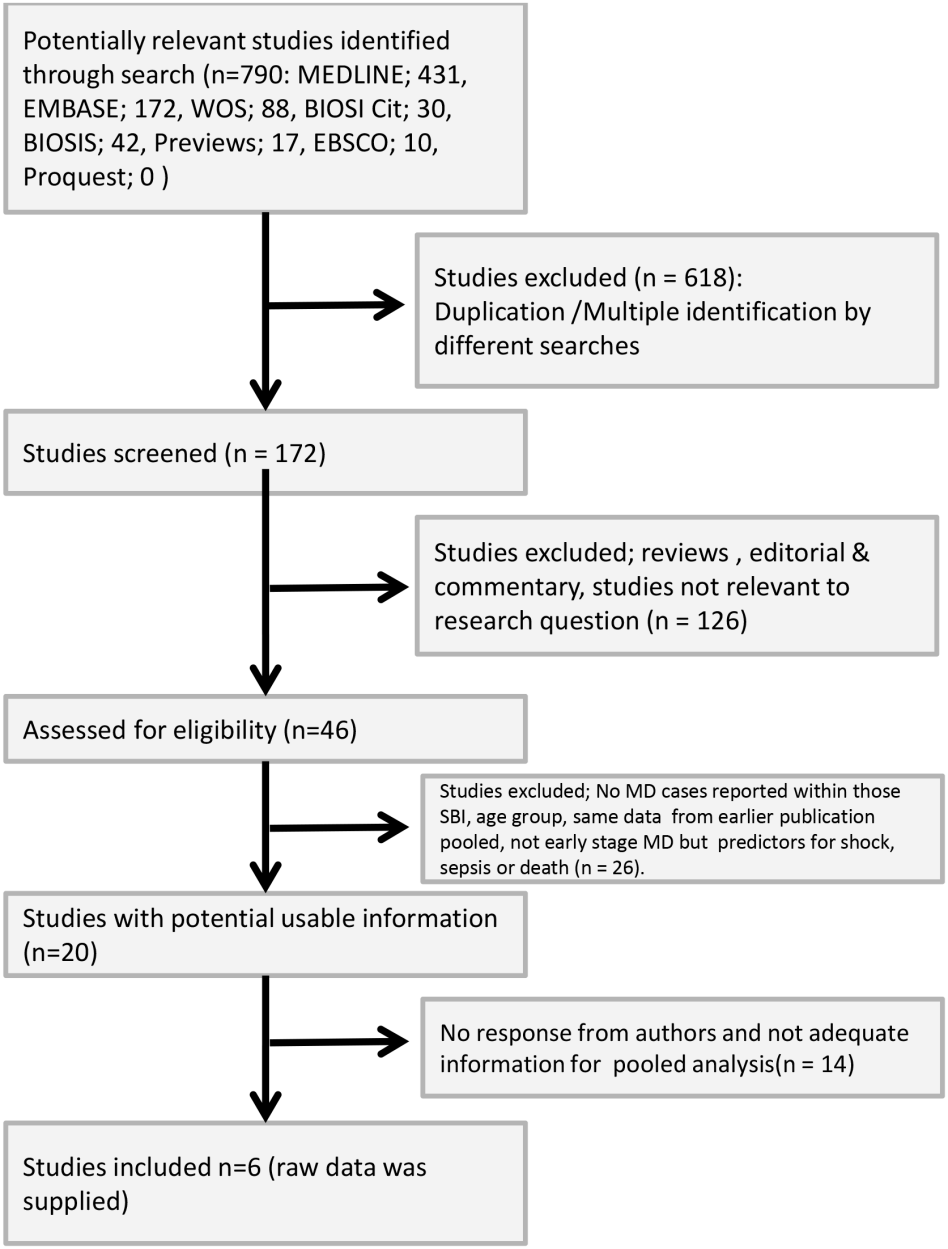
Selection of studies included in meta-analysis.

Of the 790 studies identified and screened 46 citations were selected for full text review. While 20 studies were deemed relevant for inclusion only six authors [19,21,32–35] provided sufficient raw data on PCT and/or CRP and WCC levels with corresponding microbiological results confirming MD and non-MD cases to conduct a pooled individual patient level analysis of independent studies (Fig 2 and Table B in S1 File). These six studies [19, 21, 32–35] reported on 881 children with FWS and provided the following data: 672 results for PCT, 518 for CRP and 592 for WCC (517 matched CRP and WCC results). Unpublished data from a Doctoral thesis [19], approved by the local Research Ethics Committee and supported by a research grant from The Royal Belfast Hospital for Sick Children (RBHSC), was relevant and was included in this selection.

### Quality of included studies

**Fig 3:**
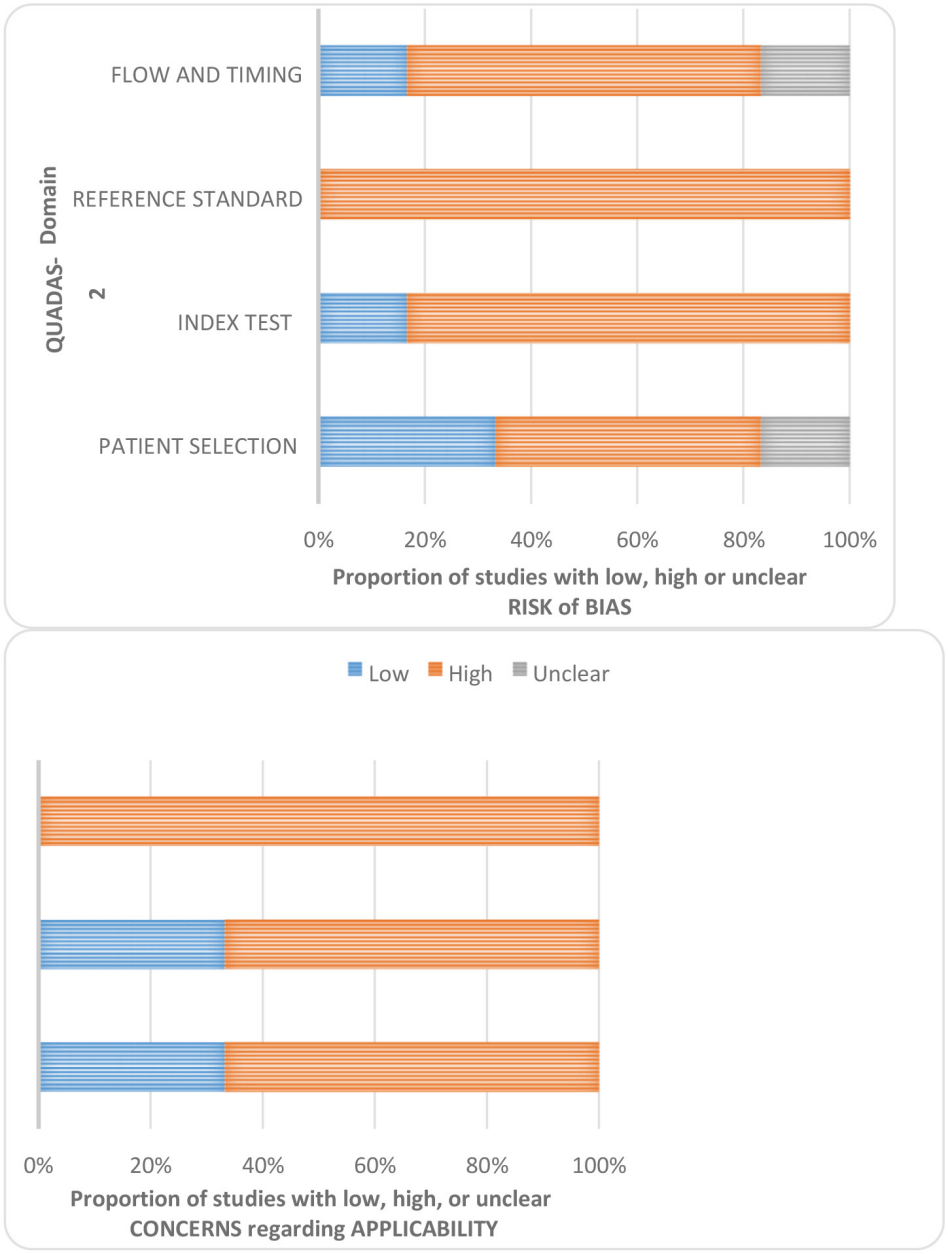
QUADAS-2 results.

In general, the quality of included studies was high (Fig 3). The data from the six studies involved children with FWS, provided adequate descriptions of the diagnostic tests and reported microbiological blood/CSF culture and/or PCR for reference test confirmation of MD. Two of six studies were focussed on SBIs in ED [33,34] and three [19, 21,35] focused on the MD care pathway within paediatric EDs. Sources of heterogeneity included the study sample size and the prevalence of MD (S1 Fig). In two of the six studies higher MD prevalence was related to the operation of a specific care pathway in the paediatric ED for patients with suspected MD [19, 21] and a local outbreak of MD in the community [35]. Table 1 summarises the characteristics of chosen studies.

**Table 1:** Summary of the characteristics of chosen studies and MD events published.

Study Ref.	Setting	Presentation	Age	Exclusion	Prevalenceb	PCT assaya	PCT cut-off (ng/ml)	Sensb	Specb% (95%CI)	Study Outcome
32	Hospital	Admission to hospital suspected meningitis	1 mnth to 14 yrs	n/a	0·22(0·09–0·43)	PCT-LIA	0.5	83 (60–99)	57 (34–77)	Elevated PCT levels in children with suspected meningitis suggests SBI
21	Paediatric Hospital	fever, rash, illness	1 mnth- 16yrs	n/a	0·64(0·55–0·72)	PCT-LIA	2	94 (85–98)	93 (80–98)	PCT is a more sensitive predictor for MD than CRP and WCC
33	Paediatric ED	Fever <12hr	1 mnth- 12yrs	n/a	0·46(0·31–0·62)	PCT-LIA	2	75 (72–99)	63 (41–82)	Distinguishing SBI from viral & localized,PCT was more sensitive than CRP for indication of SBI
19	Paediatric ED	Fever, rash, signs of meningitis, suspected meningitis	<15 yrs	n/a	0·37(0·23–0·52)	PCT-Q	1.01	83 (58–96)	74 (56–87)	PCT as predictor of early MD was better than CRP and WCC
35	Paediatric ED	Non-specific fever	<14 yrs and 14–40 yrs	UTI	0·68(0·59–0·76);<14 yrs•)0·029(0·01–0·03);adults	PCT-Q-LUMI	2;for children, 0•5;for adults	94 (75–99)	84 (83–87)	PCT as a diagnostic indicator for MD in children with non-specific fever
34	Paediatric ED	No identified source of fever after history taking and physical examination	1–36 mnths	UTI as a sub-group	0·003(0·0001–0·022)	PCT-Kryptor	0.2	100 (5–100)	68 (63–74)	CRP, PCT and WBC had similar diagnostic properties and superior to clinical evaluation in predicting SBI in children of 1–36 months•

Abrev. ED Emergency Department

^**a**^Assays made by BRAHMS GmbH (Hennigsdorf, Germany)

^b^Based on individual study diagnostic cut-off as publishedx

### Optimal diagnostic threshold

The test thresholds applied in the selected studies were not always for MD detection but to indicate the presence of SBI. The optimal diagnostic test thresholds for early MD detection were determined as 1·93ng/ml for PCT, 28 mg/l for CRP and 16 x 10^9^ /l for WCC. The Area Under Curve (AUC) for ROC plots for individual tests at these thresholds demonstrated that the PCT test with AUC = 0.95 (95% CI; 0.93 to 0.97) out-performed both CRP, AUC = 0.83 (95% CI; 0.79 to 0.87), and WCC, AUC = 0.67 (95% CI; 0.61 to 0.72) (S2 Fig)

### Meta-analysis

**Fig 4:**
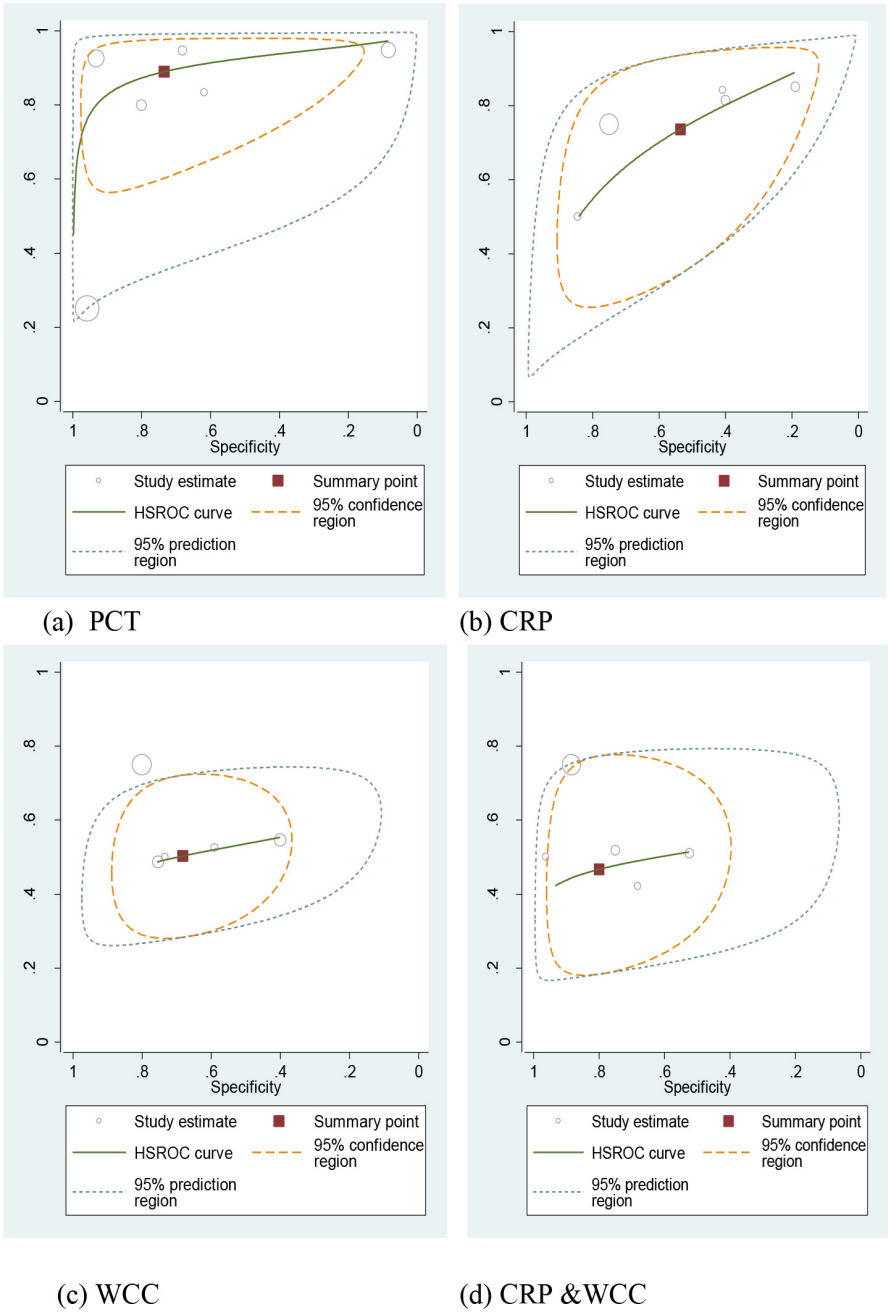
HSROC plots for selected studies for PCT, CRP and WCC individual tests and CRP with WCC.

Values for sensitivity, specificity, positive likelihood ratio (PLR), negative likelihood ratio (NLR) and odd ratio (OR) of the summary point from HRSOC plots for each test (Fig 4) are presented in Table 2. These show PCT as the most accurate test (sensitivity 89%, 95%CI 76–96%; specificity 74%, 95%CI 40–92%) for early MD compared to CRP and WCC. PCT has the best PLR (3.4, 95%CI 1.2–9.3) and most likely to suspect MD (RR of MD 4.71 95% CI 1.9–11.9) in a child with FWS compared to the CRP test (RR = 1.7, 95% CI 1.0–2.9) and WCC (RR = 1.4, 95% CI 0.9–2.3) (S3 Fig). In clinical practice, the existing tests CRP and WCC are normally used together as indicators for MD. Raised values of both (CRP+ WCC+) with non-specific symptoms would be viewed as a standard approach in distinguishing possible MD [12] when awaiting confirmatory microbiological results. To reflect this pragmatic use of both tests the *combined* performance of CRP and WCC, at optimal cut-offs levels was evaluated (Table 2). The CRP and WCC combined test had a sensitivity of 47% (95%CI 32–62) and specificity 80% (95%CI 64–90).

**Table 2:** Meta analysis of studies; summary point statistics of HSROC analysis for PCT, CRP and WCC diagnostic tests^a^.

Diagnostic Test	Sensitivity		Specificity		PLR^a^		NLR^b^		OR^c^		Prevalence
	%	95% CI	%	95% CI		95% CI		95% CI		95% CI	
**PCT**	89	76–95	74	40–92	3.4	1.2–9.3	0.2	0.07–0.3	22.5	5.7–87.8	0.3
**CRP**	74	52–88	54	31–75	1.6	1.1–2.4	0.5	0.3–0.9	3.2	1.4–7.5	0.2
**WCC**	50	40–61	68	54–79	1.6	1.0–2.4	0.7	0.6–0.9	2.2	1.1–4.2	0.2
**Combined CRP & WCC**	47	32–62	80	64–90	2.3	1.2–4.6	0.7	0.5–0.9	3.5	0.4–8.9	0.2

^a^using optimal diagnostic cut-offs

^b^ conventional positive likelihood ratio (PLR)

^c^ conventional negative likelihood ratio (NLR) ^d^ diagnostic odds ratio (OR)

#### Sub-group analysis

The effect of the age (of febrile children) on the test performance was explored at optimal cut-offs. The overall estimated relative risk (RR) of PCT indicating MD (RR 13.2; 95% CI 4.3–40.5) was greater than that of CRP (RR 3.0; 95% CI 0.8–11.1) and WCC (RR 1.6; 95% CI 1.2–2.1). Heterogeneity across age groups was not significant for WCC (I^2^ = 0%) and moderate for PCT (I^2^ = 52.5%) tests but was significant (I^2^ = 89.6%) for the CRP test. The PCT test for distinguishing prodromal MD appeared more accurate in very young (1–12 months of age) febrile children (sensitivity 95%, specificity 91%, positive likelihood ratio (PLR) 10) (S4 Fig, Table C in S1 File). Combined CRP and WCC had a greater PLR in children aged 5–9 years (sensitivity 58%, specificity 76%, PLR 3.8).

### Cost-effectiveness analysis

#### Base case

The decision analytic model was populated with the sensitivity and specificity data derived from the HSROC analysis summary point estimates, applying optimal cut-offs for each test (Table 2). In keeping with the findings of better diagnostic accuracy for PCT, the corresponding ICER for the base case was -£8,137.25 (US-$13,371.94) per correctly treated patient (Table 3). The negative value is a result of PCT + standard care being more effective and costing less than standard care i.e. it is the dominant strategy. Thus, introducing the PCT test and correctly treating more patients, despite an additional outlay of £11 (US $18.08) per test, would result in cost savings (from the payer perspective). These savings were largely attributable to improvements in the diagnostic test’s sensitivity and specificity compared to the combined CRP and WCC tests, resulting in fewer patients being incorrectly treated (due to false positive CRP/WCC diagnoses) or treatment delays among those with false negative CRP/WCC results (Tables D and Ein S1 File).

**Table 3:** Results of base case cost-effectiveness analysis of test strategies for diagnosis of MD.

Test	Cost (C)	Incremental Cost	Effectiveness (E)	Incremental Effectiveness	C/E	ICER
**Standard care**	£3476.88		0.734		£4736.89	
**PCT + standard care**	£3061.88	-£415.00	0.785	0.051	£3900.48	-£8137.25

#### Sensitivity analysis

The one-way threshold sensitivity analyses explored uncertainty in the thresholds used for the base case analysis. The results of the sensitivity analyses showed little variation across the ranges tested for the sensitivity and specificity parameters for the PCT, CRP and WCC tests (Table F in S1 File). The incremental cost-effectiveness ratios remained <-£2,000 per correctly treated patient with the exception of when the threshold PCT approached 0.2ng/ml. Thus, the sensitivity analyses indicate an improvement in diagnostic performance that is translated into cost savings. One-way sensitivity analyses were also conducted using the HSROC statistics derived following the exclusion of the study outlier. Results were essentially unchanged because the ICER indicated that PCT testing was the dominant diagnostic strategy.

## Discussion

The NICE clinical guidelines (CG102) for the management of bacterial meningitis and meningococcal septicaemia in children and young people recommend non-specific laboratory tests including CRP and WCC as being useful investigations for those presenting with FWS [12]. However, there were no recommendations concerning PCT testing. This led us to re-examine the research on the performance of PCT as a marker for early MD. We used decision modelling to compare short-term costs and benefits of using a PCT test with the recommended CRP and WCC for children with FWS presenting at ED.

In the case of early MD detection, it is desirable for a test to have high test sensitivity to reduce the chance of a missed diagnosis of potentially fatal MD. Many have demonstrated the PCT test to be more accurate for predicting invasive bacterial infections [36–45] sepsis [46, 47] and so ruling-out serious bacterial infection [28,48–50]. Within these past studies, a range of diagnostic thresholds has been used for the PCT test. After applying a diagnostic test threshold of 2 ng/ml as an indicator of possible MD previous authors found the sensitivity of the PCT test to range from 94 to 100% and specificity 84 to 100% [33, 35]. A limitation of the PCT test is that it will not distinguish between SBIs but a child presenting with FWS and an elevated PCT level is more likely to have a SBI with more severe outcomes. It is important to emphasise that alongside the clinician’s judgement, the PCT test is a useful indicator of MD when combined with standard laboratory tests and clinical symptoms. The rapid rise of PCT levels in response to a fulminant SBI such as MD make it a more valuable test in an ED setting.

Careful consideration is required in regard to the threshold level used to indicate different serious infections as it is a source of heterogeneity in test accuracy studies [51]. We were able to determine optimal cut-off levels for MD of each test, among children who presented at ED with FWS, by pooling the provided raw data. These diagnostic thresholds were compared to available literature and discussed with clinicians to ensure they were plausible and appropriate thresholds for meaningful decision analysis. When different thresholds have been employed, pooling data from a number of studies [52] will clearly affect the estimated sensitivity and specificity (known as the threshold effect). HSROC analysis was thus employed as the most appropriate method to determine the overall accuracy of a diagnostic test from data spanning 6 studies, using the optimal thresholds for each test. Although we selected similar studies, for example, for suspected disease, when and what PCT assay was used for suspected MD, substantial heterogeneity was apparent in our meta-analysis. Unrecorded and demographic differences may account for this in addition to variation in disease prevalence [53]. The 14 studies excluded due to no availability of raw data were similar to those 6 studies included in that they also took place in middle to high income countries (Europe n = 11, US n = 1, New Zealand n = 1 and Saudi Arabia n = 1), investigated patients with fever and a temperature ≥38°C, had blood tests and MD reference test taken on admission to paediatric emergency department (n = 9), hospital ward (n = 4) or ICU (n = 1), included children of varied age, with eight studies including infants only (≤ 3 years of age) and six studies including those up to 16 years of age, and finally all studies described the PCT test, MD reference test and other blood tests taken (CRP or WCC). The median incidence of the confirmed MD (n = 8), for these 14 studies that could not be included in the meta-analysis, was 5.2% (range 0.3–13%). A strength of this review was that all studies recorded appropriate robust reference tests for confirmation of MD. Although diagnostic accuracy may have been compromised by some study bias or demographic variation, the PCT test had greater sensitivity for suspected MD. At the same time, its higher specificity also implies fewer false positive results.

The better sensitivity of the PCT test also provided the basis for a more cost-effective test than the currently recommended CRP and WCC tests for the detection of early stage MD in children with FWS. A recent FEVER study found that for 3,893 febrile children, many presenting at hospital EDs, the total WCC failed to detect the most common SBI across a range of ages with a sensitivity, specificity and ROC AUC of 47%, 76% and 0.68 respectively [54], very similar to that found here. For the current review, overall test accuracy was superior for PCT when compared either with combined use of CRP and WCC or as individual tests. However, specificity for the PCT test and for combined CRP and WCC were similar. From the meta-analysis of pooled data across age groups the PCT test was a good biomarker in infants (1 month to 1 year of age): this is clinically very important as MD is more common in very young children and more difficult to diagnose in the prodromal stages. Recent interest has been in those under the age of three. A study of 226 febrile children 36 months old or younger who presented to four EDs with suspected SBI found PCT to be a more accurate biomarker than traditional screening tests for identifying young febrile infants and children with serious SBIs [55]. In this case the area under ROC curve for PCT as a test for SBIs was higher in comparison at 0.80 (95% CI 0.71 to 0.89).

Despite its superior performance it is reasonable to ask if the PCT test alone would offer a cheaper point of care option delivering quicker results. Our decision analytic model provided a clear indication of the cost-effectiveness of adding the PCT test to standard care. However, we should exercise some caution before concluding that these findings support the introduction of PCT testing into routine clinical practice. Firstly, we only considered the immediate impact of each diagnostic strategy, with the associated short-term costs and benefits. Secondly, the cost-effectiveness for each diagnostic strategy was defined as the cost per correctly treated patient. Further research is recommended to extend the analysis with a Markov model that could incorporate the impact on longer term quality-adjusted life years to calculate cost per QALY, in line with current guidance [56]. Nevertheless, our analysis suggests that the PCT test would offer savings and benefits by expediting the correct treatment for more patients and therefore reducing risks of further complications in the long term.

The difficulty in diagnosing MD in the early stages in young children is widely acknowledged. Its distinction from viral infection and other presentations is crucial for appropriate and prompt management. The consequence of not diagnosing MD early is a significant increase in morbidity and mortality. Conversely, if a diagnosis of MD is made incorrectly, some children may be unnecessarily treated with intravenous antibiotics leading to potential side-effects such as anaphylaxis or increasing antibiotic resistance.

### Conclusion

In summary, an optimal PCT level of >1.93 ng/ml could indicate early stage MD in paediatric patients presenting with FWS more quickly (in less than 1 hour) than currently recommended tests. Even when used with conventional tests it has the potential to be more cost-effective and can contribute to better antibiotic stewardship [57, 58].

## Supporting Information

S1 Fig Galbraith and Funnel plots for PCT test. (TIF)

S2 Fig ROC plots for pooled raw data for PCT, CRP and WCC tests. (TIF)

S3 Fig Forest plot for PCT test showing relative risk (95%CI). (TIF)

S4 Fig Forest plots for tests to detect prodomal MD in children with FWS of different age-groups. (TIF)

S1 File Search strategy for systematic review carried out (Table A) . Summary of diagnostic statistics for each study for each test PCT, CRP, WCC and CRP & WCC (Table B). Performance of PCT, CRP and WCC diagnostic tests in age groups pooled calculated from raw data. ^a^positive likelihood ratio (PLR) ^b^negative likelihood ratio (NLR) (Table C). Unit costs of hospital care (£ Sterling 2010–2011) (Table D). Costed clinical pathways for each patient group option*. (* These costs relate to standard care (WCC & CRP). For the intervention arm (PCT + standard care), each clinical pathway has an additional cost of £11 for the inclusion of the additional test.) (Table E). One way threshold sensitivity analysis (Table F).
(DOCX)

S1 PRISMA Checklist PRISMA 2009 Checklist for study. (DOC)
