# Supplementary figures and images for: Clinical and Cost-Effectiveness of Procalcitonin Test for Prodromal Meningococcal Disease–A Meta-Analysis
Source: PLoS One. 2015 Jun 8;10(6):e0128993. doi: 10.1371/journal.pone.0128993 (PMC4459795; doi:10.1371/journal.pone.0128993)

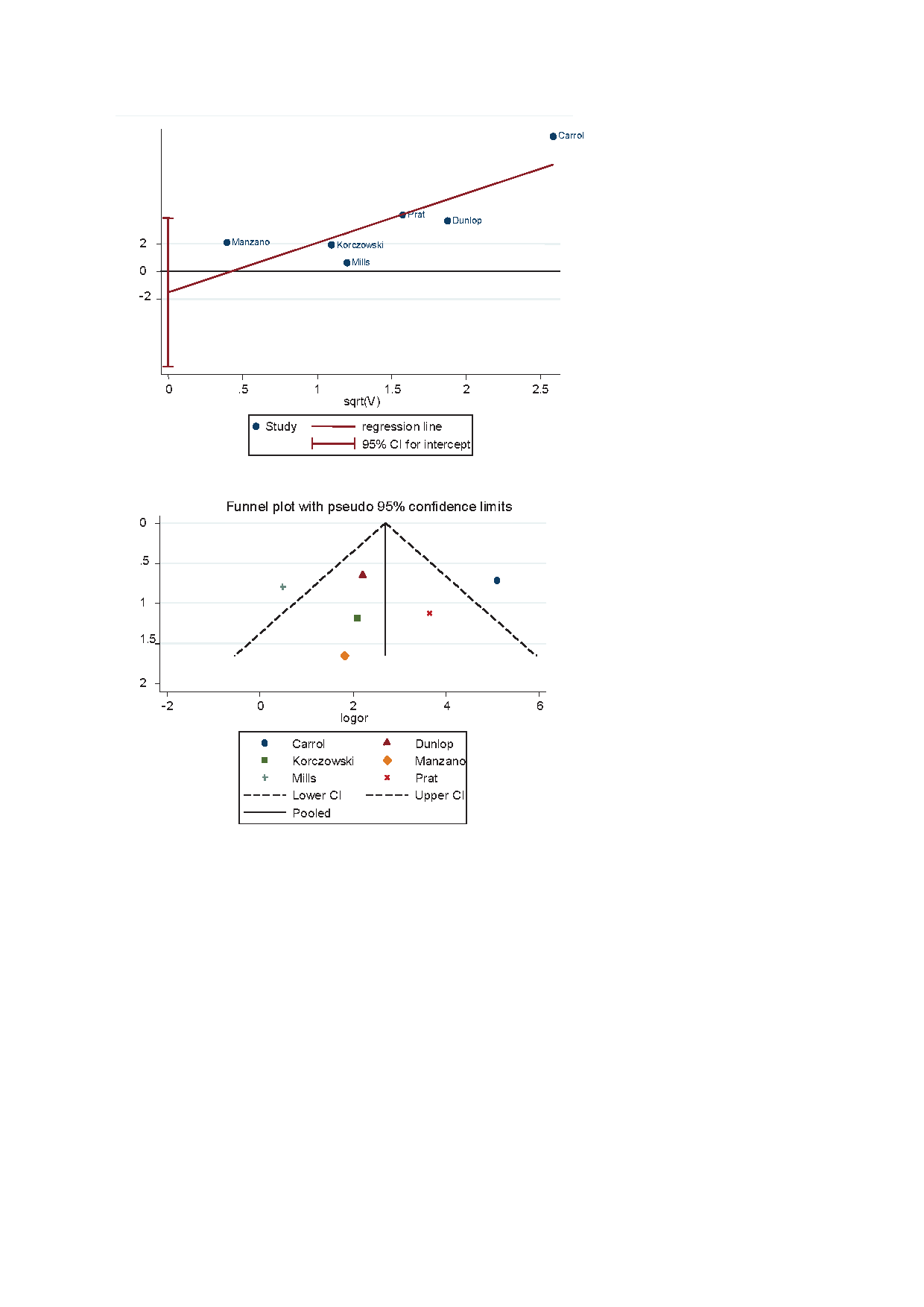

Supplement: S1 Fig — (TIF) [file pone.0128993.s001.tif]

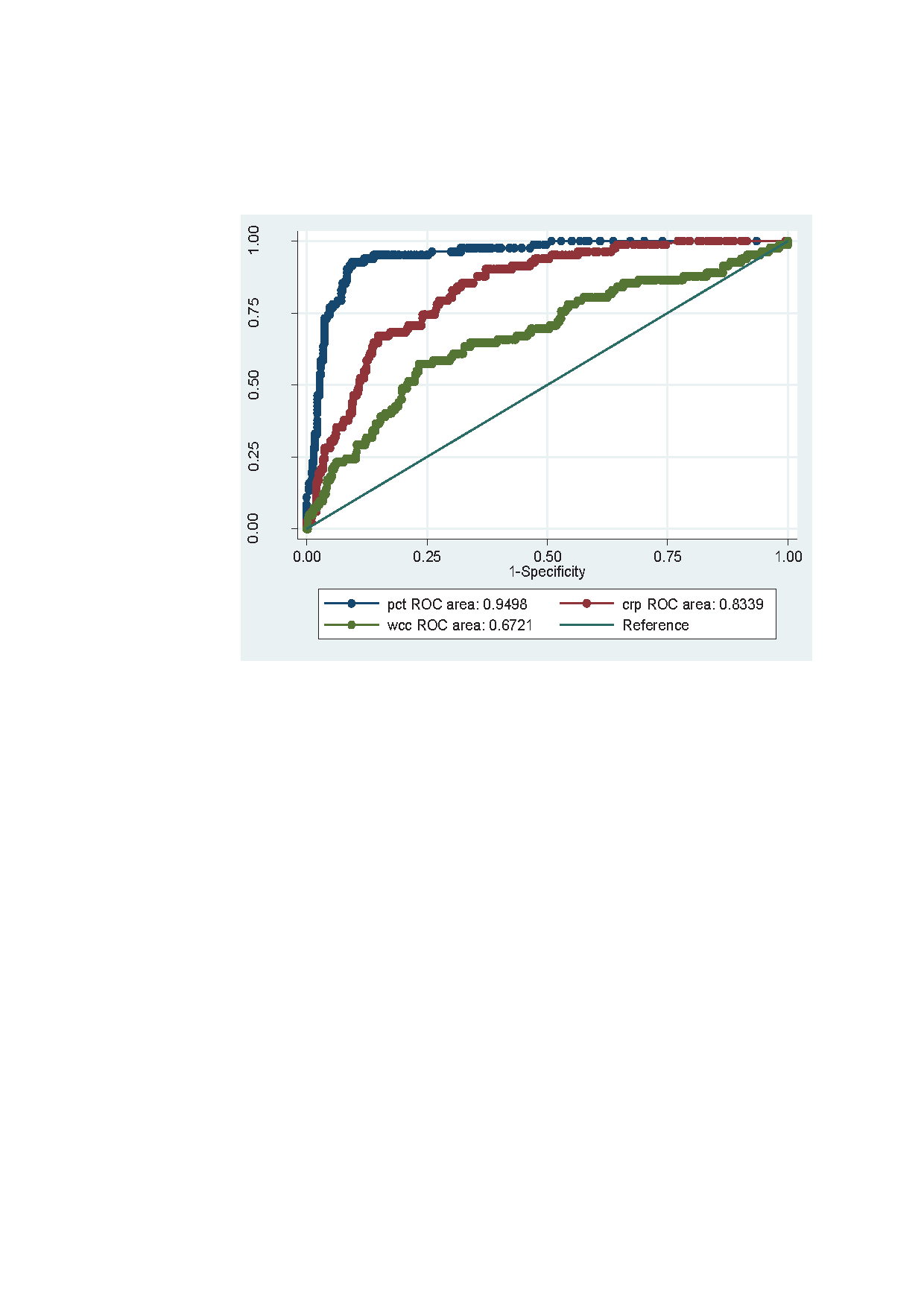

Supplement: S2 Fig — (TIF) [file pone.0128993.s002.tif]

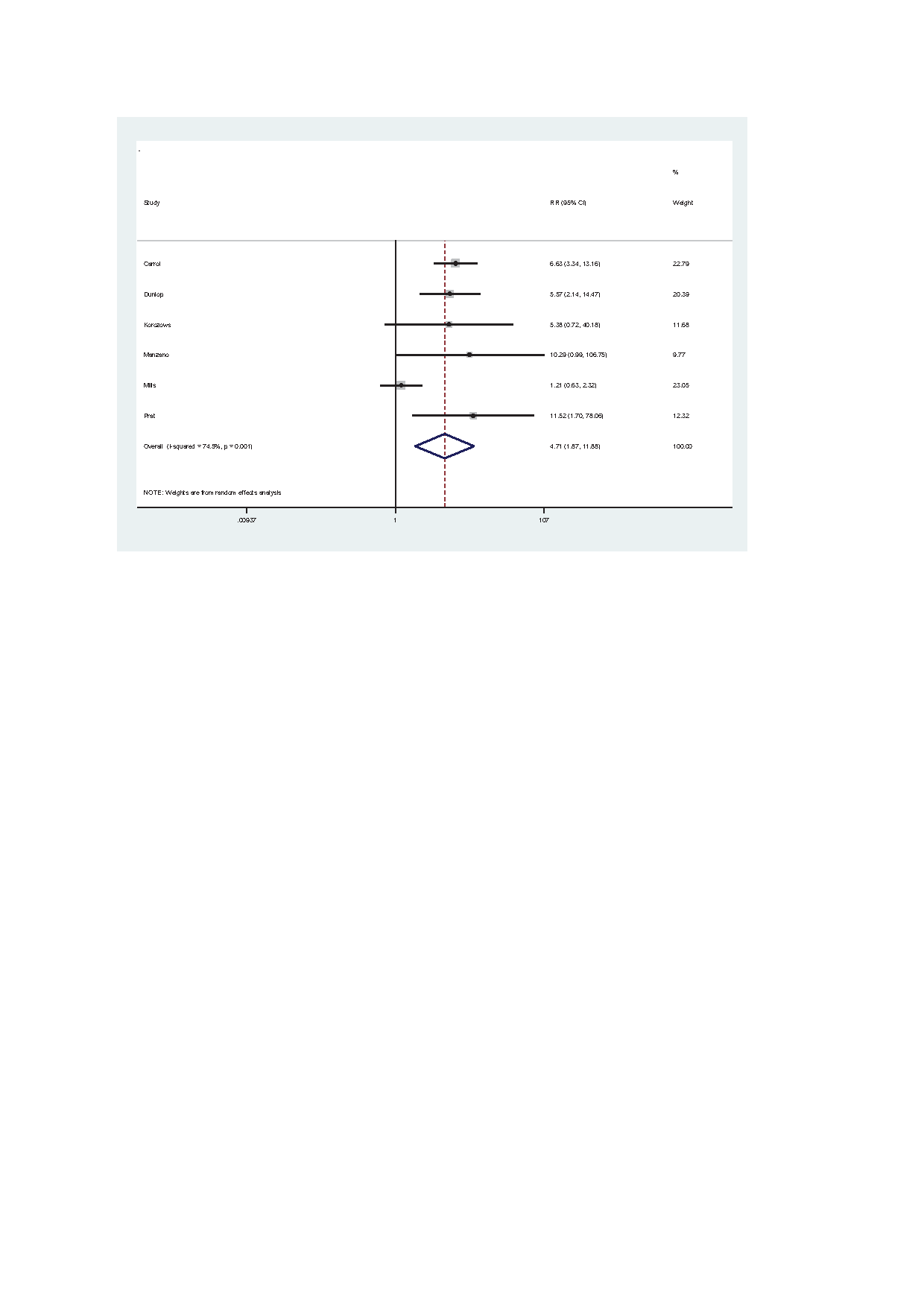

Supplement: S3 Fig — (TIF) [file pone.0128993.s003.tif]

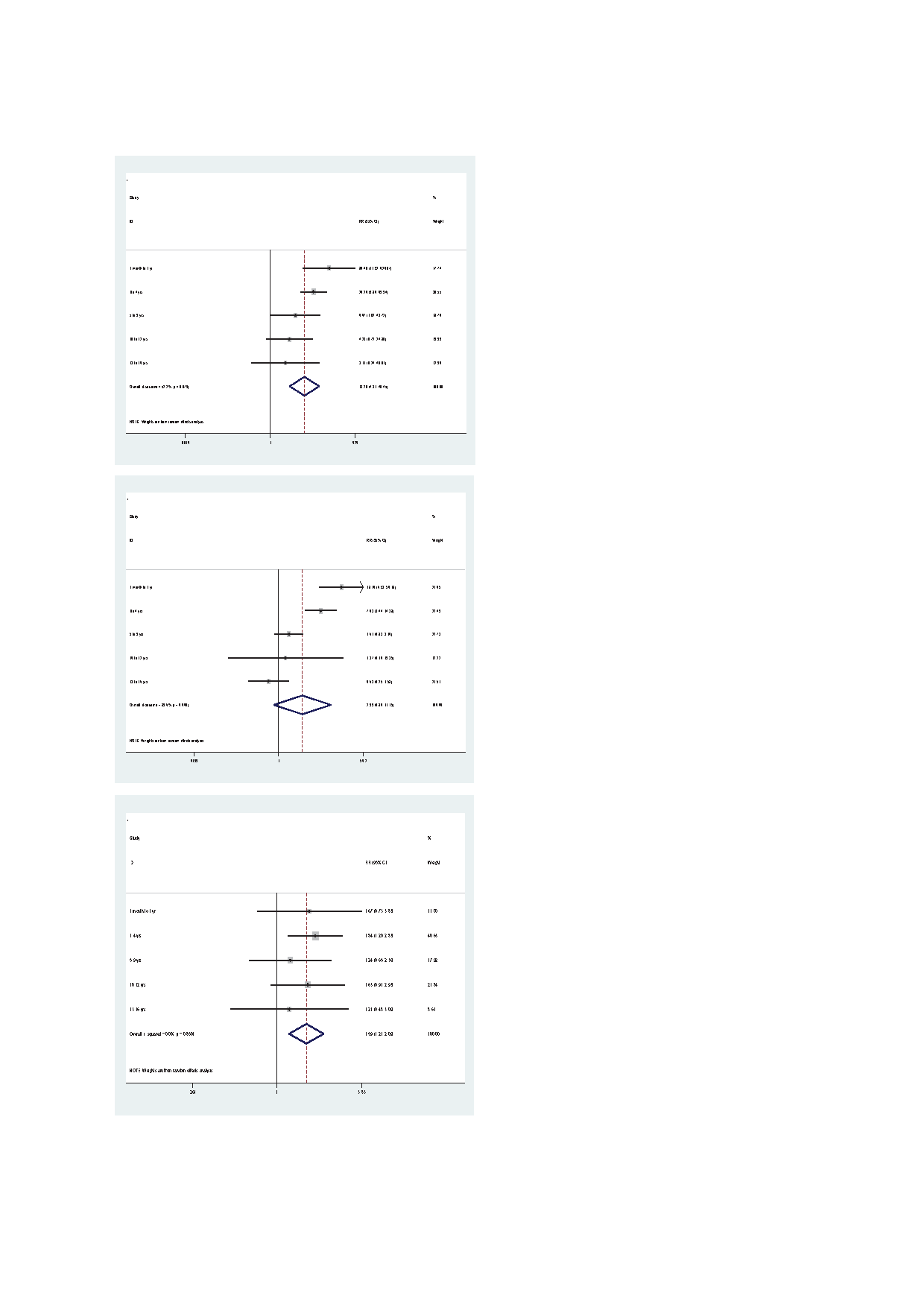

Supplement: S4 Fig — (TIF) [file pone.0128993.s004.tif]
