# Supplementary material for: Clinical and Cost-Effectiveness of Procalcitonin Test for Prodromal Meningococcal Disease–A Meta-Analysis
Source: PLoS One. 2015 Jun 8;10(6):e0128993. doi: 10.1371/journal.pone.0128993 (PMC4459795; doi:10.1371/journal.pone.0128993)
Supplement: S1 File — . Summary of diagnostic statistics for each study for each test PCT, CRP, WCC and CRP & WCC (Table B). Performance of PCT, CRP and WCC diagnostic tests in age groups pooled calculated from raw data. apositive likelihood ratio (PLR) bnegative likelihood ratio (NLR) (Table C). Unit costs of hospital care (£ Sterling 2010–2011) (Table D). Costed clinical pathways for each patient group option*. (* These costs relate to standard care (WCC & CRP). For the intervention arm (PCT + standard care), each clinical pathway has an additional cost of £11 for the inclusion of the additional test.) (Table E). One way threshold sensitivity analysis (Table F). (DOCX) [file pone.0128993.s005.docx]

**Table A. Search strategy for systematic review carried out**

| MEDLINE® Search August 2011 |
| --- |
| 1 mening*.mp.  2 (Neisseria adj1 meningitidis).mp.  3 (Meningococcal adj1 meningitis).mp.  4 (Meningococcal adj1 disease).mp.  5 exp Meningococcal Infections/ or exp Meningitis, Meningococcal/ or exp Neisseria meningitidis/ or Meningococcal s?epticaemia.mp.  6 (bacteri* adj1 mening*).mp.  7 1 or 2 or 3 or 4 or 5 or 6  8 PCT.mp.  9 (procalcitonin adj1 test).mp.  10(procalcitonin adj5 marker).mp.  11 (serum adj1 procalcitonin).mp.  12 biomarker*.mp.  13 exp Biological Markers/ or exp Calcitonin/ or procalcitonin.mp.  14 8 or 9 or 10 or 11 or 12 or 13 or 14 or 15 or 16  15exp Intensive Care Units, Pediatric/ or exp Hospitals, Pediatric/ or p?ediatric*.mp.  16 exp Child/ or child*.mp.  17PICU*.mp.  18 15 or 16 or 17  19 7 and 14 and 18  20 limit 19 to humans |

**Table B. Summary of diagnostic statistics for each study for each test PCT, CRP, WCC and CRP&WCC**

**(a) PCT**

| Author ref | Sensitivity (% 95% CI) | Specificity (% 95% CI) | PPV (95% CI) | NPV (95% CI) | MD Prevalence (95%CI) | |
| --- | --- | --- | --- | --- | --- | --- |
| 21 | 93 (85-97) | 93(80-98) | 0.97(0.90-0.99) | 0.85(0.71-0.93) | | 0.68(0.60-0.76) |
| 19 | 80(56-93) | 80(63-91) | 0.70(0.47-0.86) | 0.88(0.70-0.96) | | 0.36(0.24-0.50) |
| 31 | 83(37-99) | 62(39-81) | 0.38(0.15-0.68) | 0.93(0.64-1.0) | | 0.22(0.09-0.43) |
| 33 | 25(0-91) | 96(93-98) | 0.04(0-0.33) | 0.99(0.97-1.0) | | 0.007 (0.001-0.027) |
| 34 | 95(87-0.98) | 8 (2-24) | 0.69(0.59-0.77) | 0.43(0.12-0.80) | | 0.68(0.59-0.76) |
| 32 | 95(72-100) | 68(45-85) | 0.72(0.50-0.87) | 0.94(0.68-1.0) | | 0.46(0.31-0.62) |

**(b) CRP**

| Author ref | Sensitivity (95% CI) | Specificity (95% CI) | PPV (95% CI) | NPV (95% CI) | MD Prevalence (95% CI) | |
| --- | --- | --- | --- | --- | --- | --- |
| 21 | 50 (0-100) | 86(71-95) | 0.08(0-0.56) | 0.99(0.86-1) | | 0.02(0.00-0.14) |
| 19 | 81 (61-93) | 40(26-56) | 0.45(0.31-0.60) | 0.78(0.56-0.92) | | 0.38(0.27-0.50) |
| 31 | 75(9-100) | 75(69-.92) | 0.02(0.00-0.09) | 0.99(0.98-1.0) | | 0.007(0.00-0.03) |
| 33 | 85(71-93) | 19(6-43) | 0.70(0.56-0.81) | 0.36(0.12-0.68) | | 0.7(0.57-0.79) |
| 34 | 84(60-96) | 41(21-63) | 0.55(0.36-0.73) | 0.75(0.43-0.93) | | 0.46(0.31-0.62) |

**(c) WCC**

| Author ref | Sensitivity (95% CI) | Specificity (95% CI) | PPV (95% CI) | NPV (95% CI) | MD Prevalence (95% CI) | |
| --- | --- | --- | --- | --- | --- | --- |
| 21 | 50 (0-100) | 74(57-86) | 0.05(0-0.4) | 0.98(0.83-1) | | 0.02(0.00-0.14) |
| 19 | 49 (32-65) | 75(62-85) | 0.53(0.35-0.70) | 0.72(0.60-0.82) | | 0.36(0.27-0.46) |
| 31 | 75(9-100) | 80(79-75) | 0.02(0.00-0.11) | 0.99(0.98-1.0) | | 0.007(0.001-0.03) |
| 33 | 55(43-66) | 40(24-58) | 0.67(0.54-0.78) | 0.29(0.17-0.43) | | 0.7(0.59-0.77) |
| 34 | 53(29-75) | 59(37-79) | 0.53(0.29-0.75) | 0.59(0.37-0.79) | | 0.46(0.31-0.62) |

**(d) CRP&WCC**

| Author ref | Sensitivity (95% CI) | Specificity (95% CI) | PPV (95% CI) | NPV (95% CI) | MD Prevalence (95% CI) |
| --- | --- | --- | --- | --- | --- |
| 21 | 50 (0-1) | 96(83-100) | 0.25(0-0.91) | 0.75(0.09-1) | 0.02(0.00-0.14) |
| 19 | 52 (32-71) | 75(59-86) | 0.56(0.350.75) | 0.72(0.560.84) | 0.38(0.27-0.50) |
| 31 | 75(9-100) | 88(84-92) | 0.04(0.000.18) | 0.99(0.98-1.0) | 0.007(0.0010.03) |
| 33 | 51(37-65) | 52(30-74) | 0.71(0.53-0.85) | 0.31(0.17-0.49) | 0.70.58-0.80) |
| 34 | 42(21-66) | 68(45-85) | 0.53(0.27-0.78) | 0.58(0.37-0.76) | 0.46(0.31-0.62) |

**Table C. Performance of PCT, CRP and WCC diagnostic tests in age groups pooled calculated from raw data.** ^a^positive likelihood ratio (PLR) ^b^negative likelihood ratio (NLR)

| Diagnostic test | Age Group | Sensitivity | Specificity | PLR^a^ | NLR^b^ | Prevalence |
| --- | --- | --- | --- | --- | --- | --- |
| PCT | 1 month-12months | 95 | 91 | 10 | 0.1 | 0.1 |
|  | 1yr -4yrs | 92 | 89 | 8.3 | 0.1 | 0.2 |
|  | 5 yrs- 9 yrs | 94 | 53 | 2.0 | 0.1 | 0.5 |
|  | 10 yrs-12 yrs | 92 | 67 | 2.8 | 0.1 | 0.7 |
|  | 13yrs -16 yrs | 100 | 13 | 1.2 | 0.0 | 0.5 |
| CRP | 1 month-12months | 89 | 76 | 3.6 | 0.2 | 0.1 |
|  | 1yr -4yrs | 84 | 67 | 2.5 | 0.2 | 0.2 |
|  | 5 yrs- 9 yrs | 68 | 55 | 1.5 | 0.6 | 0.5 |
|  | 10 yrs-12 yrs | 100 | 38 | 1.6 | 0.0 | 0.6 |
|  | 13yrs -16 yrs | 83 | 7 | 0.9 | 2.5 | 0.4 |
| WCC | 1 month-12months | 30 | 81 | 0.9 | 0.9 | 0.1 |
|  | 1yr -4yrs | 42 | 76 | 0.8 | 0.8 | 0.3 |
|  | 5 yrs- 9 yrs | 62 | 48 | 0.8 | 0.8 | 0.5 |
|  | 10 yrs-12 yrs | 57 | 83 | 0.5 | 0.5 | 0.7 |
|  | 13yrs -16 yrs | 81 | 25 | 0.8 | 0.8 | 0.5 |
| CRP&WCC | 1 month-12months | 29 | 90 | 2.7 | 0.1 | 0.1 |
|  | 1yr -4yrs | 45 | 85 | 3.0 | 0.7 | 0.7 |
|  | 5 yrs- 9 yrs | 58 | 76 | 3.8 | 0.5 | 0.5 |
|  | 10 yrs-12 yrs | 63 | 83 | 2.8 | 0.1 | 0.1 |
|  | 13yrs -16 yrs | 66 | 27 | 0.9 | 1.3 | 1.3 |

**Table D. Unit costs of hospital care (£ Sterling 2010-2011)**

| **Description of hospital spell** | **Currency Code** | **Currency Description** | **National Average Unit Cost** |
| --- | --- | --- | --- |
| **Accident and emergency investigation leading to admitted** | VB01Z-VB09Z | Accident and emergency Weighted Average | £151 |
| **Hospital stay for diagnosis** | PA20B | Fever, unspecified without CC (non-elective, short stay mean 1day) in child aged 18 years and under | £470 |
| **Hospital stay for fever, unspecified** | PA20B | Fever, unspecified without CC (non-elective, long stay excess bed day) | £425 |
| **Hospital stay for fever, unspecified** | PA20B | Fever unspecified without CC (non-elective, long stay mean 2.21 day) in child aged 18 years and under | £1,229 |
| **Hospital stay for MD, unspecified** | AA22B | Non-Transient Stroke or Cerebrovascular Accident, Nervous system infections or Encephalopathy without CC (non-elective, long stay 6.26 day) | £2,334 |
| **Hospital day case stay for MD, unspecified** | AA22B | Non-Transient Stroke or Cerebrovascular Accident, Nervous system infections or Encephalopathy without CC (day case) | £497 |
| **Paediatric follow-up appointment** | 420 | Paediatrics. Consultant-led follow-up non-admitted face-to-face | £166 |
| **Hearing test** | 254 | Paediatric Audiological Medicine | £154 |
| **PICU bed day** | XB01Z-XB07Z | PICU Weighted Average | £1,433 |
| **PCT test** |  | Brahms UK, Biomarkers Clinical Diagnostics Division. | £11 |

**Table E. Costed clinical pathways for each patient group option.***

| **Patient group** | **Clinical pathway components** | **Total cost** |
| --- | --- | --- |
| **TRUE POSITIVE**  Severe | 1) Presentation to accident and emergency, admitted  2) Admission to PICU (mean stay 4 days)  3) Discharged  4) Paediatric outpatient follow-up appointment.  5) Paediatric audiological medicine outpatient appointment (hearing test) | £6,203 |
| **TRUE POSITIVE**  Moderate | 1) Presentation to accident and emergency, admitted  2) Hospital spell for unspecified meningococcal disease (long stay)  3) Day case for unspecified meningococcal diseases (to complete antibiotics)  4) Discharged  5) Paediatric outpatient follow-up appointment.  6) Paediatric audiological medicine outpatient appointment (hearing test) | £4,297 |
| **FALSE NEGATIVE**  Severe | 1) Presentation to accident and emergency, admitted  2) Hospital spell for unspecified meningococcal disease (short stay)  3) Discharged  4) Transfer to PICU  5) Admission to PICU (mean stay 6 days)  6) Discharged/dies[query in red below] | £11,867 |
| **FALSE NEGATIVE**  Moderate | 1) Presentation to accident and emergency, admitted  2) Hospital spell for unspecified meningococcal disease (long stay)  3) Discharged | £2,485 |
| **TRUE NEGATIVE**  Severe | 1) Presentation to accident and emergency, admitted  2) Admitted to PICU (mean stay 3 days)  3) Hospital spell for unspecified fever (long stay)  4) Discharged | £5,679 |
| **TRUE NEGATIVE**  Moderate | 1) Presentation to accident and emergency, admitted  2) Hospital spell for unspecified fever (long stay and 4 excess bed days)  3) Discharged | £3,081 |
| **TRUE NEGATIVE**  Mild | 1) Presentation to accident and emergency, admitted  2) Hospital spell for unspecified fever (long stay)  3) Discharged | £1,380 |
| **FALSE POSITIVE**  Mild | 1) Presentation to accident and emergency, admitted  2) Hospital spell for unspecified fever (long stay)  3) Discharged | £1,380 |

* These costs relate to standard care (WCC & CRP). For the intervention arm (PCT + standard care), each clinical pathway has an additional cost of £11 for the inclusion of the additional test.

**Table F. One way threshold sensitivity analysis**

| Diagnostic test and threshold | ICER (£/correctly treated patient | |
| --- | --- | --- |
|  | Standard care | PCT + standard care |
| PCT max 2ng/ml | Dominated | -£21,672.78 |
| PCT min 0.2ng/ml | Dominated | £2,329.79 |
| Combined CRP&WCC Max. 40mg/l & 15x10^9^/l | Dominated | -£44,097.78 |
| Combined CRP&WCC Min. 17.7mg/l & 14.1 x10^9^/l | Dominated | -£2,348.54 |
